# Supplementary material for: Associations of maximum and reactive strength indicators with force–velocity profiles obtained from squat jump and countermovement jump
Source: PLoS One. 2022 Oct 21;17(10):e0276681. doi: 10.1371/journal.pone.0276681 (PMC9586393; doi:10.1371/journal.pone.0276681)
Supplement: Attachment — Submitted filename: TO AUTHORS.docx [file pone.0276681.s002.docx]

Congratulations for your work. Some concerns should be resolve before publication.

The short title doesn't talk about jump performance.

In your introduction you should talk about the term that you want to resolve with your research. Please rewrite this section giving more information about the F-v measurement importance.

In the first appearance should be stretch-shorting cycle (SSC). Review all the abbreviations throughout the manuscript and remember that abstract section is independent.

Line 78 What was the reason to have this range of hours?

Line 88: Delete mean ± SD

Line 107: rest (60-90 s)

Line 115: How and when did you estimate the individual RM?

Line 116: At the end of the warm up you were close to RM, it was this weigth used at the initial attemps?

Line 136: Why absolute value and not RM percentage?

Line 143: under the same environmental conditions

Line 203: What was the result of normality test?

Line 259: Explain here your hypothesis in relation with your results.
